# Supplementary material for: Are carbon emissions trading and green financial instruments synergistic? -Comprehensive quantitative research based on content analysis
Source: PLoS One. 2024 Mar 7;19(3):e0298601. doi: 10.1371/journal.pone.0298601 (PMC10919601; doi:10.1371/journal.pone.0298601)
Supplement: S1 Data — (ZIP) [file pone.0298601.s001.zip › Data/地级市Z_F工作报告.zip!/╔╜╢1⁄2/║╩╘≤╩╨/└┤╫╘╣1⁄2╓┌║┼í╛┬φ┐╦╩2╛▌═°í┐_2009.docx]

各位代表:

　　现在,我代表市人民政府向大会作政府工作报告,请予审议,并请市政协委员和其他列席人员提出意见。

　　一、2008年工作回顾

　　过去的一年,全市人民在中共菏泽市委的坚强领导下,深入落实科学发展观,围绕实现“四个发展”,强力推进“四大基地一大产业”建设,狠抓招商引资、项目建设、工业运行、城市建设、“三农”工作、改善民生等重点工作,较好地完成了市十七届人大一次会议确定的各项任务。

　　(一)国民经济平稳较快发展。实现地区生产总值822亿元,增长15.6%,高于全省3.5个百分点。完成地方财政收入50.4亿元,增长19.8%。税收占地方财政收入的比重达到77%,同比提高0.9个百分点。金融机构存款余额704亿元、贷款余额484亿元,分别比年初增加134亿元和81亿元。城镇居民人均可支配收入11581元,农民人均纯收入4584元,均比上年有较大增长。节能降耗、环境保护完成了上级下达的目标任务。

　　(二)经济结构进一步优化。三次产业比调整为23.8:50.5:25.7,二三产业比重提高2.7个百分点。规模以上工业企业达到1481家,增加176家;完成增加值352亿元,增长25.7%;实现主营业务收入1356亿元,增长41.4%;利税128.5亿元,增长48.5%。新增省级以上企业技术中心3家,达到18家。实现高新技术产业产值269亿元,增长50%。达驰电气获得省政府质量奖。服务业发展迅速,完成增加值211亿元,增长20.3%;实现社会消费品零售总额405亿元,增长23.5%;完成进出口总额9.94亿美元,增长16.6%。

　　(三)“四大基地一大产业”建设初见成效。以大项目建设为重点,大力推进“四大基地一大产业”建设,完成投资249亿元,占千万元以上在建项目投资的83%。铁雄新沙一期150万吨焦化、圣世化工60万吨MCC、达驰电气500千伏超高压节能变压器和洪业化工14万吨己二酸等72个过亿元项目竣工投产;华润电厂一期、东明石化120万吨高硫重油综合利用、巨润优质浮法玻璃、舜亦新能源等110个过亿元项目开工建设。文化产业快速发展,创建中国优秀旅游城市取得显著成效。在“四大基地一大产业”项目的带动下,全市千万元以上在建项目完成投资300亿元,增长22%。其中亿元以上项目270个,完成投资180亿元,分别增长28%和25%。

　　(四)农村经济全面发展。粮食总产101亿斤,增产2.75亿斤,单产、总产均创历史新高。棉花总产5.77亿斤,略有增加。营造片林23.9万亩。肉类总产52.4万吨,增长6.6%。新增规模以上龙头企业60家,达到540家;新增农民专业合作组织204个,达到907个。农田水利会战成效显著,洙赵新河上游清淤圆满完成,东鱼河截蓄导工程进展顺利,4大灌区改造全面实施,完成水利建设投资10.5亿元。改造农村公路1230公里,新建沼气池1.5万个,新增自来水受益人口100万人。认真落实强农惠农政策,兑付各类补贴资金9.7亿元。

　　(五)城市建设力度加大。城市建设工程多、投入大、标准高、速度快,规划建设管理水平不断提高。市区开工城建工程70余项,完成投资33.8亿元。大剧院、演武楼、市垃圾处理厂等工程已经或即将投用;赵王河南段景观建设、新世纪公园建设等工程进展加快;新改建城区道路42公里,菏泽监狱搬迁工作顺利启动,长江路高标准改造基本完工;洙水河、外护堤河、赵王河等河道清淤截污全面竣工。市区冬季新植树木30万棵。全市新开工房地产面积190万平方米,花城经济适用房交付使用,全市4477户低收入家庭获得政府住房补贴。城区供水、供热、供气覆盖范围进一步扩大。单县千干山公园、巨野县人民路改造等一批县城重点建设项目完工,基础设施不断完善。环卫体制改革继续深化,城乡环境明显改观。327国道改造已经完工,德商高速南段正式通车,鄄城黄河公路大桥、洙水河航道等重点基础设施工程进展顺利。

　　(六)和谐社会建设成效显著。新增城镇就业11万人。完成各类农民培训22万人次,新增劳务输出20万人。新增社会保险参保人数17万人次,总数达到183万人次,征缴各类保费16亿元,增加2亿元。城镇居民基本医疗保险全面启动,参保人数达到23万人。城市低保人均月补差提高到100元,农村低保人均年补差达到480元,实现了应保尽保。新农合参合率达到93%,社区卫生服务覆盖人口达到80%以上。消除中小学危房25.6万平方米,城区10所中小学建设基本完成;教育教学质量又有新提高,普通高校本科录取人数有较大增长。公共文化服务工程建设取得新进展,非物质文化遗产入选数量居全省首位。十一届全运会武术散打比赛筹备工作进展顺利。广播、电视人口综合覆盖率分别达到98%和95%。人口计生工作取得新进展,人口自然增长率控制在4.78‰。支援北川抗震救灾和恢复重建工作成绩显著,捐赠款物累计9519万元,过渡房援建任务率先完成,受援乡小学、卫生院等5个项目建设进度较快。奥运安保工作圆满完成,社会治安和安全生产形势持续稳定。市场管理得到加强,产品质量和食品药品安全专项整治成效明显。人防、气象、地震、档案、史志、民族宗教、外事侨务、对台工作、民兵预备役、老龄、妇女儿童和残疾人等各项事业都有了较快发展。

　　各位代表!2008年是极不寻常的一年。在经济形势复杂多变的不利环境下,能够取得这样的成绩,确实来之不易。这是我们在市委正确领导下,深入贯彻科学发展观、认真落实省委、省政府决策部署的结果,是市人大、政协和社会各界有效监督、大力支持的结果,是全市人民齐心协力、众志成城、扎实苦干的结果。在此,我代表市人民政府,向全市广大干部群众,向各民主党派、工商联、人民团体和社会各界人士,向驻菏部队、武警官兵和中央、省属单位,向所有关心支持菏泽发展的朋友们,表示诚挚的敬意和衷心的感谢!

　　在充分肯定成绩的同时,我们也清醒地看到,经济社会发展中还存在不少困难和问题,主要是:受国际金融危机影响,企业效益下滑,工业运行困难增多;返乡农民工明显增多,农产品价格全面回落,农民持续增收难度加大;大项目建设受到的资金、土地、环保等因素制约越来越大;地方财力紧张,财政增收面临很大压力;社会事业发展滞后,社会保障能力不强;不稳定因素增多,维护稳定任务艰巨;政府及其部门的工作作风、服务质量和行政效率还有待进一步改进和提高,等等。对此,我们一定高度重视,采取有力措施,切实加以解决。

　　二、2009年目标任务

　　在新的一年里,我们既面临国内外经济形势急剧变化带来的严峻挑战,更面临国家保增长、扩内需给我市“四大基地一大产业”建设带来的难得机遇。我们必须振奋精神,坚定信心,抢抓机遇,化危为机,迎难而上,共克时艰,继续推动全市经济社会更好更快发展。政府工作的总体要求是:深入贯彻党的十七大、十七届三中全会精神,认真落实科学发展观,紧紧抓住中央扩大投资、拉动内需的机遇,按照抓投资促建设、抓运行保增长、抓调整促升级、抓招商促开放、抓改革增活力、抓民生促和谐的指导思想,坚定不移谋发展,千方百计促和谐,全力以赴保稳定,努力促进全市经济社会科学发展、和谐发展、跨越发展、更好更快发展。

　　全市经济社会发展的主要预期目标是:地区生产总值增长13%;规模以上工业企业主营业务收入增长21%,利税增长21%;千万元以上项目完成固定资产投资365亿元;社会消费品零售额增长18%;外贸进出口增长10%;外商直接投资增长8%;地方财政收入增长12%;新增城镇就业9万人,登记失业率控制在4.6%以内,人口自然增长率控制在6.3‰以下;城镇居民人均可支配收入和农民人均纯收入均增长9%;万元GDP能耗下降5.46%;二氧化硫、化学需氧量分别削减1.5%和2.1%。

　　围绕实现上述目标,着力抓好以下几项工作:

　　(一)抓住国家扩大投资机遇,强力推进大项目建设。按照在建项目抓投产达效、已批复项目抓开工建设、拟新上项目抓审核批复的要求,狠抓大项目建设目标责任制落实,努力形成“竣工投产一批、开工建设一批、准备开工一批、策划储备一批”的良性循环。一是抓好中央新增投资项目建设和争取工作。对列入2008年中央新增投资的项目,着力抓好资金配套,切实加快项目建设进度,确保3月底前形成实物工作量。同时,抓住今明两年国家扩大内需和投资机遇,进一步加大保障性安居、农村民生、基础设施、医疗卫生、教育文化、节能减排、生态建设、自主创新和结构调整等领域资金争取工作力度,确保争取政策性资金占全省的10%。二是全力推进大项目建设。狠抓项目资金到位、工程质量和建设进度。重点抓好铁雄新沙焦化二期、华润电厂、舜亦新能源等75个在建投资过亿元项目的建设;突出抓好赵楼煤矿、东明石化120万吨高硫重油综合利用、洪业化工10万吨环己酮、玉皇化工5万吨顺丁橡胶和开发区玉皇化工20万吨苯乙烯等124个大项目的竣工投产;着力抓好日东输油管道、郓城120万吨焦化联产、开发区聚隆能源10万吨甲醇、赵楼煤矸石综合利用电厂一期等101个大项目的开工建设。三是加大项目储备力度。紧紧围绕“四大基地一大产业”建设,搞好现代能源化工、高新技术产业、商贸物流、文化旅游和基础设施等领域项目的策划论证,选择一批资源条件好、产业链条长、国家政策鼓励发展的项目,争取得到国家和省的审批,各类项目储备确保300亿元。力争列入省重点的项目个数占全省10%以上。四是着力抓好招商引资。以资源招商、产业招商和项目招商为重点,加强专业招商队伍建设,采取“大批量、小团队”等组织形式,到珠三角、长三角、青烟威等重点区域招商引资,保证常年有500个以上团组在外招商。继续办好国际牡丹花会和中国林产品交易会,搭建招商引资平台,吸引更多客商来菏泽投资兴业。积极做好境外上市工作,已进入上市程序的5家企业尽快择机上市,另有10家以上企业进入上市程序。五是切实强化园区建设。抓住关键环节,强化服务措施,提高工作效率,努力化解制约大项目建设的瓶颈,确保大项目引得进、建得快、早投产、快达效。着力抓好园区内道路、供电、供气、供排水、铁路专用线等基础设施的完善,同时加快金融、物流、商贸、居住等服务设施的建设。扎实做好煤化工“一地三园”规划的报批工作。力争园区千万元以上项目完成投资240亿元,其中基础设施投资10亿元。

　　(二)全力抓好工业运行,不断提高发展质量和效益。一是加强企业资金管理,确保企业资金链条不断裂。加大对现有企业的信贷扶持力度,对目前产品有市场、经营有效益、发展有前景、运转正常但遇到暂时财务困难的企业,重点给予信贷支持和政策扶持。每个县区都要建立2000万元以上的中小企业贷款风险周转金,专项用于帮助暂时还贷困难的企业按时还贷。二是突出抓好企业生产经营管理。以精细化管理为抓手,广泛开展“练内功、挖潜力、增效益”活动,重点加强资金、成本管理,积极推行5S管理、定额管理等先进管理经验,着力从原料购进、加工生产到产品销售各个环节加强管理,降低成本费用,提高盈利能力。加强对企业两项资金占用的分析和研究,不断提高企业产销衔接水平,最大限度压缩库存,加快资金周转,提高企业效益。组织企业大力开展职工培训,不断提高职工队伍素质。三是大力开拓市场。引导企业加快产品结构调整,扩大适销对路产品生产,积极创新现代营销模式,坚持让利不让市场,努力保持和提高市场占有率。推动工商联手,组织开展好地产品进超市活动。四是加快企业自主创新。以发展“新特优”为方向,引导企业通过技术改造新上改扩建项目,重点抓好100个技改项目。搞好200家规模企业技术中心建设和产学研联合,积极搭建技术创新、科技合作平台。力争东明玉皇化工技术中心进入国家级企业技术中心,单县湖西王集团、鄄城华意化工完成省级技术中心认定,20家企业完成市级认定。加快发展高新技术产业,着力抓好30户重点高新技术企业,高新技术产业产值达到380亿元,增长35%以上。五是加强工业运行调度。继续加大对60户重点企业的扶持力度,落实好各项鼓励发展政策,打造一批全国知名企业。实行工业运行指挥部工作机制,重点调度年主营业务收入亿元以上的260户企业,及时协调解决影响生产经营的突出矛盾和问题。对停产半停产企业,组成帮扶工作组,深入企业现场办公,切实解决实际问题,促其正常生产经营。

　　(三)加快发展农村经济,扎实推进新农村建设。认真贯彻落实十七届三中全会精神,着力搞好城乡统筹,积极发展现代农业,促进农业增效、农民增收、农村繁荣。一是扎实推进农业结构调整。积极争取国家“新增千亿斤粮食生产能力建设”等项目,切实抓好中低产田改造,确保粮食总产稳定在100亿斤以上。搞好平原林业产业化示范区创建工作,新增造林面积14万亩。加快发展林下养殖、水产养殖,林下养殖面积达到65万亩,畜禽饲养量达到3500万头(只),水产养殖面积达到33万亩。发挥政策引导作用,加大农机投入,农业综合机械化水平提高到70%。深入实施“科技兴农”战略,着力完善农业科技创新和推广体系,加快新技术、新成果的转化,加强市县农产品质量监测中心建设,认真做好禽流感、美国白蛾和东亚飞蝗、灰飞虱等重大病虫害防控工作。二是大力发展农业产业化经营。突出抓好龙头企业培育、合作经济组织和标准化生产基地建设。加大扶持力度,确保30家重点企业和52家支撑企业主营业务收入增长15%以上;力争规模以上龙头企业新增60家,达到600家;优质农产品生产基地新增100万亩,达到550万亩;绿色产品认证新增10个,达到40个;无公害产品认证新增15个,达到60个;农民专业合作经济组织新增300个,达到1200个以上。三是努力促进农民增收。积极发展农村二三产业,搞活多种经营,拓宽农民增收渠道。多形式、多途径开展实用技术培训,抓好有组织劳务输出,确保农民务工收入稳定增加。四是着力加强农村基础设施建设。继续组织好农田水利会战,筹划建设一批河道拦蓄工程,重点推进巨野大野水库、郓城南赵楼水库、东鱼河截蓄导工程等项目建设,基本完成单县浮岗水库和曹县太行堤水库除险加固,切实抓好闫潭、苏泗庄等六大灌区改造,提高抗旱防汛能力。进一步加快农村路、水、电建设,新增改水受益人口60万人,自来水普及率达到58%以上,新改建农村公路1500公里,新建沼气池2.4万个。五是深化农村各项改革。认真执行强农惠农政策,及时将补贴资金和市县配套资金落实到位。抓好土地流转有形市场试点,推动土地依法有序流转。加快推进畜牧兽医管理体制改革,健全动物疫病防控体系。加大水利建设力度,发展以水库水源地为中心的规模化集中供水。加快农村信用社发展和改革,力争获准筹建4家农村合作银行。大力发展民营经济,积极构建区域特色经济发展格局。

　　(四)繁荣商贸流通和文化旅游业,推动服务业快速发展。坚持把发展服务业作为拉动内需、扩大消费、促进就业和结构调整的重要举措。一是大力发展商贸流通业。以打造区域性商贸物流基地为目标,加快东明石化物流园区、巨野煤化工物流园区等十大物流园区建设。做好菏泽口岸保税物流园区筹建工作,力争年内开工建设。积极扶持菏泽交通物流、华瑞物流、三信物流等物流企业快速发展。重点抓好中国牡丹商贸城、曹县庄寨林产品大市场等独具特色的专业批发市场建设。实现专业批发市场交易额560亿元,增长30%以上。通过招商引资、兼并联合等形式,组建大型现代商贸流通企业。继续实施“万村千乡市场工程”和“农产品进超市工程”,切实组织好家电下乡活动,把连锁、超市等新型流通形式向农村延伸,繁荣农村市场,扩大农村消费。进一步完善菏泽公路集疏运中心场站体系,新建续建客货场站7处,改造农村客运站37处,客货运周转量分别达到6340万人和5750万吨。二是大力发展对外贸易。充分利用出口退税率调整政策,积极引导企业加快结构调整步伐,努力扩大高附加值产品出口。加大国际市场开拓力度,积极开辟东盟、中东、南美、非洲等新兴市场。引导企业抓住当前有利时机,扩大原油、大豆、木材等原材料和关键设备进口。加强出国劳务基地建设,扩大劳务输出规模。三是加快发展文化旅游业。巩固和完善创优成果,争取尽快通过国家验收。有效整合牡丹、戏曲、书画等旅游资源,打造“一城三乡一生态”旅游品牌和一批独具特色的旅游景点。以传统农家风情、农活体验为载体,创建一批乡村旅游示范点。接待国内外游客420万人次,实现旅游收入30亿元。四是积极发展新兴服务业。加快发展电子政务、电子商务等信息服务业,培植家政、托幼、养老、保健等名牌社区服务业,支持鼓励各类中介机构和行业协会健康发展。

　　(五)加大城市和基础设施建设力度,加速推进城市化进程。

　　坚持大投入、多开工、快建设、出精品的思路,打造亮点工程、精品片区和标志性地段。一是加强城镇规划设计。进一步加快城乡规划编制,市区和县城控制性详细规划覆盖率及乡镇总体规划覆盖率力争达到100%,村庄规划覆盖率力争达到50%。进一步放开规划设计市场,实行方案征集制度,着力提升片区、项目和单体设计水平。二是集中力量推进重点工程建设。上半年全面完成长江路、大剧院、演武楼、赵王河景观带等续建工程。启动并基本建成洙水河公园、胜利广场,开工建设规划展览馆,实施烈士陵园、口岸搬迁。完善城市路网结构,打通西安路、和平路南段等断头路,改造部分城区街巷道路,力争开工建设大学路、东南水区路网等一批城市道路。实施道路、河道及老城区管道截污,新建污水泵站5处,新增污水管网73公里。深入推进“增花增绿”工程建设,丰富“花城林海”城市特色。下大力抓好供水、供热、供气工作,新铺设供热管网7公里,增加采暖面积100万平方米;新建供气管道12公里,发展天然气用户4000户,努力改善居民生活条件。推进县城重点工程建设,完善基础设施,改善县城形象,提升城市功能。三是大力发展城市房地产业。加快市区绿城国际、华英嘉园、龙燕阳光城等大型房地产开发项目建设,努力打造精品楼盘。全面推开长江路中部、太原路南部、和平路北部、双河路东部等多个片区综合开发。力争市区新开工房地产面积200万平方米,其中开工高层80栋、面积140万平方米。完成“城中村”改造试点5个。四是全面提升城市管理水平。推行精细化管理模式,加强净化、绿化、亮化、美化及市政设施管理,开展城乡环境综合整治,以良好面貌迎接全运会。实施城市公交专项规划,加快农村客运发展。完善拆迁补偿办法,严厉打击违法搭建行为。五是加快重大基础设施建设。大力推进德商高速北段、鄄城黄河公路大桥、洙水河航道等工程建设,开工建设东新高速,做好菏枣高速、菏泽至曲阜机场专用线、巨郓河航道和郓城港口等项目前期工作。借助京九、新菏兖日铁路电气化改造机遇,切实抓好菏泽火车站改扩建工程,建设巨郓矿区、东明石化、市开发区煤化工业园等一批地方铁路专用线。

　　(六)强化财政增收节支,努力做好金融工作。一是发挥好财政杠杆作用。加大地方财政对重点财源的支持,集中力量保运行、保发展、保增收。面对国家实施积极财政政策、扩大投资的机遇,积极做好项目资金争取工作,并及时足额落实配套资金。认真落实增值税转型、税收减免等一系列税制改革政策,确保及时执行到位。坚持依法治税管费。不断完善税源控管体系,强化社会综合治税,严厉打击偷逃骗税行为,确保应收尽收。积极推进财税资源优化重组,引导企业实施二、三产业分离,增加地方财政收入。严格执行“收支两条线”制度,提高非税收入管理质量。规范政府公共资源有偿使用管理,做好国有资产收益收缴工作。二是加强公共财政建设。严格按照保工资、保运转、保民生、保稳定的要求,合理安排预算,强化预算约束。大力压缩一般性支出。对购车用车、会议经费、公务接待、出国经费等支出实行零增长。继续深化部门预算、国库集中支付、政府采购、会计委派等项改革,加强财政资金监管,防止截留、挤占、挪用资金等行为。三是进一步搞活资金融通。坚持政银企三方联动,着力破解资金难题。引导企业与金融机构加强合作,增进了解,增强信任,实现共赢。认真落实“十一五”银企战略合作协议,重点抓好签约项目的资金到位。大力支持各类金融机构发展,积极吸引股份制银行来菏设立分支机构。鼓励和引导金融机构加大对骨干企业和重点项目的支持,加大对民生工程、“三农”、节能减排等扩大内需项目的支持,积极创新金融产品,切实搞好银团、社团贷款的组织实施。全年新增贷款规模力争达到150亿元。建立健全贷款担保体系,扩大现有担保公司规模,搞好小额贷款公司试点,规范发展民间融资。加强社会信用建设,严厉打击失信行为,防范和化解金融风险,打造一流的金融生态环境。

　　(七)切实抓好节能减排和土地节约集约利用,加快推动经济发展方式转变。一是大力推进节能降耗。认真执行固定资产投资项目节能评估审核制度,严格控制新上高耗能项目。继续抓好420户重点企业对标节能活动,对年耗能4000吨标煤以上的60户企业实行重点监控。强化节能技术改造,重点实施10大节能工程、100个节能技术改造项目。积极推广节能新技术,开发节能新产品,加快高耗能设备的淘汰改造。推进循环经济示范试点工作,抓好电力、煤炭、化工等重点行业循环经济发展。加强节能执法监管,对完不成节能目标的企业和县区,实行“项目限批”和“区域限批”。二是着力加强环境保护。认真落实新上项目环评和“三同时”制度,严格控制新增污染。加大环境执法监管力度,严厉打击违法排污行为。加快东西区污水处理厂和市县污水管网规划建设,提高污水收集率;鼓励企业使用中水,促进市县中水厂正常运营,确保三条主要河流出境断面水质明显改善。积极推进城区燃煤锅炉改造和达标治理,加快电力行业脱硫工程建设和粉尘污染治理,确保城区空气质量不断改善。切实加强农村环境保护工作。三是严格土地节约集约利用。坚持最严格的耕地保护制度和节约用地制度,强化用地审批管理,控制投资强度、建筑容积率,最大限度提高土地利用效率。深入开展土地违法违规专项整治行动,对达不到要求的县区实行“区域限批”。搞好新一轮土地规划修编,加大开发整理复垦力度,为大项目建设和经济社会发展提供可靠用地保障。

　　(八)着力改善民生,促进社会和谐。

　　千方百计扩大就业。加强就业培训,提高就业能力。充分发挥人力资源市场的作用,强化就业援助和服务,重点做好高校毕业生、复员军人和零就业家庭人员就业。实行企业减员报告制度,严格控制企业裁员。鼓励新开工企业和项目安置返乡农民工,引导扶持农村劳动力就地创业就业。

　　努力提高社会保障水平。以城镇基本医疗保险参保和企业养老保险征缴为重点,不断加大扩面征缴力度,完成各类保费征缴16亿元。逐步提高社会救助水平,城乡低保标准分别提高到每人每月200元和每人每年1000元,城乡大病人均救助标准分别提高到3800元和2800元。农村“五保”集中供养率稳定在70%以上。积极实施医疗、教育、减灾、慈善等救助救济。加大保障性住房建设力度,着力缓解城镇低收入家庭住房困难,全市建设经济适用房32万平方米、廉租房6万平方米。大力发展老龄、残疾人和社会福利事业。继续做好对口支援北川恢复重建工作,确保我市首批援建项目尽快建成,第二批项目年底完工。

　　优先发展教育事业。认真落实国家义务教育阶段各项政策,完善经费保障机制,严肃查处教育乱收费行为。加快农村中小学危房改造,维修改造25万平方米,全部消除D级危房,并做好“两热一暖一改”试点工作。加大城区学校规划建设力度,建成省级职教实训基地2处,各县区新建1所城区中小学。加强师资队伍建设,完善教师补充机制,强化业务培训,提升教学质量。深入实施农村支教工程,促进城乡教育均衡发展。加快菏泽学院西校区置换和二期工程建设,做好迎接本科教学水平评估工作;积极推进菏泽医专新校区、菏泽职业技术学院一期和菏泽家政职业学院二期工程建设进度。加快发展文化体育事业。认真做好市县图书馆、艺术馆、博物馆等文化基础工程规划建设工作,确保60%的乡镇建成综合文化站,1000家标准化农家书屋全部完成建设任务。实施精品工程,繁荣文艺创作。加强文化市场管理,净化城乡文化环境。巩固广播电视“村村通”工程成果,新增农村有线电视用户7万户;城区数字电视用户推广率达到60%。扎实推进“一点三线”全民健身工程建设,全力承办好十一届全运会武术散打比赛。加强文化遗产保护。积极做好史志、档案等工作。

　　大力发展卫生计生事业。进一步巩固和完善新农合制度,农民参合率达到97%以上。用足用好上级扶持政策,抓好乡镇卫生院和农村卫生体系国债建设项目的实施,全面完成2381处农村卫生室建设和29处社区卫生服务机构建设任务。加强各类传染病防治,规划内疫苗接种率达到95%以上。加强医院管理,改进医德医风,规范医疗行为,扩大限价收费范围,努力为人民群众提供安全、有效、方便、价廉的医疗卫生服务。广泛开展爱国卫生运动。切实加强组织机构建设,做好红十字会工作。严格落实人口与计划生育目标管理责任制,强化城市和流动人口管理,加快县乡计生服务站建设步伐,着力提高出生人口素质,进一步稳定低生育水平。确保全市人口出生率控制在12.8‰以内,合法生育率达到93%以上。

　　全力抓好安全生产。深入开展“安全生产责任落实年”活动,严格落实政府监管和企业主体责任,不断提升基层安全生产管理水平。强化安全生产专项整治,狠抓事故隐患排查治理,坚决遏制重特大安全事故的发生。提高极端天气和地质灾害预报预防能力,做好防灾减灾工作。进一步加强应急救援体系建设,完善预案,加强演练,最大限度减少人民群众人身伤亡和财产损失。

　　提高食品药品安全监管水平。强化政府管理责任和企业主体责任,扎实推进食品安全示范县、示范乡镇创建工作,注重从源头抓起,健全监管体制,全面加强食品药品监督管理,严厉打击食品药品制假售假行为,切实保障人民群众饮食用药安全。

　　切实维护社会和谐稳定。强化社会治安综合治理,严厉打击严重暴力刑事犯罪、农村盗抢牛羊农机具和城区“两抢一盗”等多发性侵财犯罪,完善社会治安防控体系,有效增强人民群众的安全感。坚持领导干部公开接访制度,着力解决突出信访问题,加大信访积案化解力度,及时妥善处置突发事件。扎实开展普法教育,全面推进依法治市。做好民族宗教工作,促进民族团结、宗教规范和睦。深入开展国防教育,继续推进双拥共建。

　　三、进一步加强政府自身建设

　　以开展学习实践科学发展观活动为契机,按照“四高”要求,全面加强自身建设,集中精力抓大事、抓重点、抓服务、抓落实、抓成效,全面提高政府工作水平。

　　(一)加强政策研究,破解发展难题。积极参加并搞好即将开展的“科学发展观”学习实践教育活动,坚持用中国特色社会主义理论武装头脑。系统学习、深入研究国家宏观经济政策,准确把握决策部署,及时捕捉政策信息,迅速采取应对措施,切实提高抓好落实、驾驭全局的本领,提高在危机中逆势而上、在困境中逆向崛起的本领,全力争创一流业绩,促进经济社会更好更快发展。

　　(二)切实转变作风,务求工作成效。按照市委部署,落实“四大基地一大产业”领导小组负责制、市级领导分包重点项目和联系县区等责任制度,细化各项发展指标,分解到单位,量化到个人,形成一级抓一级、一级对一级负责的目标责任体系。进一步精简会议,压缩文件,减少应酬,提高效率,坚持一线工作法,集中更多的时间和精力深入基层解决实际问题。继续坚持大项目一月一调度、一季度一观摩等行之有效的做法,加强督导,强化检查,跟踪问效,确保重点任务落到实处。健全完善督导考核机制,严格依法考核,严肃查处各类弄虚作假行为,实行真奖真罚,重奖重罚,营造真抓实干、干事创业的良好氛围。

　　(三)全面搞好服务,优化发展环境。进一步深化行政审批制度改革,精简审批事项,优化审批流程,缩短审批时间,大力提高服务效能。继续实行部门主要负责人电话公开、限时办结和服务承诺三项制度,尽心竭力为群众服务,及时帮助企业解决实际困难。全面实行部门收费由收变缴和一费制,规范入企检查。大力纠正部门和行业不正之风,严厉打击“三乱”行为,着力优化企业发展环境。

　　(四)规范政府行为,严格依法行政。严格按照法定权限和程序行使权力、履行职责,依法管理经济社会事务。自觉接受人大的法律监督和政协的民主监督,全力办好人大代表建议和政协委员提案,广泛听取各民主党派、人民团体的意见和建议。健全行政执法责任制,加强执法监督,严肃查处各类违法乱纪行为。积极推进重大事项集体决策、专家论证、社会公示制度,提高政府决策的科学化、民主化和法制化水平。加强电子政务、政府网站、市长公开电话建设,畅通信息渠道,拓展公开内容,保障人民群众的知情权、参与权、监督权。

　　(五)强化廉政建设,做到廉洁勤政。坚持“一岗双责”,认真落实党风廉政建设责任制,严格执行廉洁自律各项规定。深入开展反腐败斗争。强化审计监督和行政监察,完善工程招投标、政府采购、土地出让、产权交易以及领导干部经济责任审计等制度,加强对重要部门、重要岗位和重要环节的监管,着力构建长效监督机制,树立政府廉洁勤政的良好形象。

　　各位代表!新的一年挑战与机遇同在,困难与希望并存。化危为机,事在人为;逆境崛起,关键在干。我们坚信,在中共菏泽市委的坚强领导下,只要全市人民振奋精神,扎实苦干,攻坚克难,开拓进取,我们就一定能够夺取2009年各项工作的全面胜利,一个经济文化繁荣、文明富裕和谐的菏泽在不久的将来定会展现在世人面前!
